# Real-World Impact of Research Feedback Reports on CYP Mental Health for Families of Children With Rare Genetic Disorders and Intellectual and Developmental Disability

**DOI:** 10.1192/bjo.2024.251

**Published:** 2024-08-01

**Authors:** Harriet Housby, Ramya Srinivasan, IMAGINE ID Consortium, David Skuse, Jeanne Wolstencroft

**Affiliations:** UCL Institute of Child Health, London, United Kingdom

## Abstract

**Aims:**

Children and young people (CYP) with intellectual and developmental disabilities (IDD) of known genetic origin experience complex physical and mental health problems; IMAGINE-ID has followed a national UK cohort from childhood to early adulthood. Parents completed structured online psychiatric assessments on repeated occasions. From these assessments, semi-automated personalised reports were generated summarising each child's strengths and difficulties, in collaboration with IMAGINE ID participants and the charity UNIQUE.

We aimed to discover whether providing a structured summary of our mental health and behavioural assessments would be beneficial to families of children with rare genetic conditions and IDD.

**Methods:**

574 of the CYP's caregivers completed an online ‘impact’ survey, five years after receiving their initial report, comprising four areas of potential benefit: Quality of Care (whether the report led to an improvement in the child's quality of mental and/or physical health care); Social Impact (whether the report was used as evidence to support an EHCP, disability benefits etc.), Psychological Impact (whether it led to any change in understanding of the child's condition), and Referrals (whether the report led to a referral for Autism/ADHD etc.). We also invited qualitative feedback.

**Results:**

82% of respondents rated the reports as helpful. 35% reported they had led to an improvement in their CYP's quality of care, 24% reported social impact using the report as supporting evidence, 99% reported a psychological impact – a change in their understanding of the child, and 17% used the report to initiate a referral for an assessment of ADHD and/or autism. In our qualitative analysis, families who found the report helpful mentioned it led to ‘reflection’ on their child's condition and that it provided ‘access to benefits’. For those who did not find the report helpful, issues such as ‘it lacked professional input’ and ‘forgetting the contents’ of the report were identified.

**Conclusion:**

Personalised summary reports, based on a structured assessment of their child's behavioural, social and emotional adjustment, are valued by families of children with rare genetic conditions and IDD and can bring about tangible benefits to the child and the family's access to resources.

